# Mapping of Networks to Detect Priority Zoonoses in Jordan

**DOI:** 10.3389/fpubh.2015.00219

**Published:** 2015-10-12

**Authors:** Erin M. Sorrell, Mohammad El Azhari, Nezar Maswdeh, Sarah Kornblet, Claire J. Standley, Rebecca L. Katz, Ibrahim Ablan, Julie E. Fischer

**Affiliations:** ^1^Milken Institute School of Public Health, George Washington University, Washington, DC, USA; ^2^Jordan Field Epidemiology Program, Ministry of Health, Amman, Jordan

**Keywords:** one health, zoonotic disease, Jordan, laboratory, disease surveillance

## Abstract

Early detection of emerging disease events is a priority focus area for cooperative bioengagement programs. Communication and coordination among national disease surveillance and response networks are essential for timely detection and control of a public health event. Although systematic information sharing between the human and animal health sectors can help stakeholders detect and respond to zoonotic diseases rapidly, resource constraints, and other barriers often prevent efficient cross-sector reporting. The purpose of this research project was to map the laboratory and surveillance networks currently in place for detecting and reporting priority zoonotic diseases in Jordan in order to identify the nodes of communication, coordination, and decision-making where health and veterinary sectors intersect, and to identify priorities and gaps that limit information sharing for action. We selected three zoonotic diseases as case studies: highly pathogenic avian influenza (HPAI) H5N1, rabies, and brucellosis. Through meetings with government agencies and health officials, and desk research, we mapped each system from the index case through response – including both surveillance and laboratory networks, highlighting both areas of strength and those that would benefit from capacity-building resources. Our major findings indicate informal communication exists across sectors; in the event of emergence of one of the priority zoonoses studied, there is effective coordination across the Ministry of Health and Ministry of Agriculture. However, routine formal coordination is lacking. Overall, there is a strong desire and commitment for multi-sectoral coordination in detection and response to zoonoses across public health and veterinary sectors. Our analysis indicates that the networks developed in response to HPAI can and should be leveraged to develop a comprehensive laboratory and surveillance One Health network.

## Introduction

The emergence and spread of new pathogens is one of today’s highest global health security risks with zoonotic diseases arguably the chief contributor. Zoonoses occur at the interface of human and animal health, impacting a wide range of health services and livelihoods. Social and political issues surround their assessment and management. Zoonotic viruses, parasites, bacteria, and fungi are recognized as threats to human health and sustainable development worldwide, and are a major concern for national and international agencies (1). Significant risk factors for the emergence and rapid spread of zoonotic diseases include international travel; global trade; increasing interactions among humans, wildlife, exotic, and companion animals; human behavior; rapid microbial adaptation; changing climates and ecosystems; and changing livestock management practices (2). Domestic animals and wildlife are well-known reservoirs of many emerging infectious diseases; roughly 75% of recent emerging infections and 60% of all human pathogens are of zoonotic origin (3–6).

Although zoonotic diseases clearly present a significant threat to human and animal public health, many remain neglected due to competing priorities; for example, ministries of health are coping with growing burdens of non-communicable chronic diseases alongside existing maternal and child health needs, whereas ministries of agriculture/wildlife tend to prioritize livestock management for food production and trade. The costs of zoonoses in lives and livelihoods can be enormous. The effects of zoonoses on human health and economies have recently been underscored by notable outbreaks, such as the 2009 H1N1 influenza virus pandemic, which began in swine farms on the Mexico–US border. Unfounded fears that meat products could transmit “swine flu” led to major losses to the North American pork industry, amounting to 25 million USD per week, and the banning of importation of pigs and pork products by at least 15 countries (7). In addition to natural disease threats, several zoonoses are among agents that have the potential to cause severe health threats if accidentally or deliberately released.

Understanding zoonotic disease emergence, prevention, and control requires multi-disciplinary, collaborative basic and applied research. Communication and coordination among national disease surveillance and response networks are vital in ensuring the timely response to a public health event. Through systematic infectious zoonotic disease data collection, we can gain a better understanding of disease emergence and spread and provide mechanisms upon which to build early warning and response systems for animal and human health. Various frameworks aim to support capacity building for disease surveillance and response, including the World Health Organization’s International Health Regulations (IHR), the World Organisation for Animal Health’s (OIE) Animal Terrestrial Code and Pathway to Veterinary Services (PVS), and the Global Health Security Agenda (GHSA) (8–11). Although systematic information-sharing between the human and animal health sectors can help decision-makers detect and respond to zoonotic diseases rapidly, resource constraints, and other barriers often prevent efficient cross-sector reporting. Despite significant investments in technology, knowledge, and the availability of the frameworks and programs noted above, many countries still face significant gaps in their abilities to prevent, detect, and respond effectively to public health threats, including zoonotic diseases.

The Hashemite Kingdom of Jordan’s abilities to prevent, detect, and respond to zoonoses have been tested and strengthened over recent years, spurred by a large brucellosis outbreak nearly a decade ago and a highly pathogenic avian influenza (HPAI) H5N1 outbreak in 2006. The Ministry of Health’s (MOH) Division of Zoonotic Diseases and the Ministry of Agriculture’s (MOA) Veterinary Services have developed a strong and cooperative relationship across surveillance and laboratory sectors. Although these relationships exist, they are informal and used only in the context of response to major outbreaks or events. By mapping zoonotic disease detection, reporting, and response capacities across surveillance and laboratory systems, we sought to determine where mechanisms exist to integrate single-disease networks into national zoonotic response and to identify best practices/systems that can be applied across all priority zoonoses. Such mapping not only can help identify hotspots where zoonoses pose significant health threats but also where efforts can be focused to improve prevention, communication, and coordination across veterinary and human health.

## Materials and Methods

The methodology consisted of systematically mapping the laboratory and surveillance networks currently in place for detecting and reporting priority zoonotic diseases in Jordan. Our analysis does not include geographical mapping but rather an analysis reviewing major elements of systematic capacity building as outlined by Potter and Brough (12). We identified, collated, and then mapped the current surveillance and laboratory systems in place to detect, assess, report, and respond to zoonotic diseases using publically available reports and key informant interviews. The relevant subject matter experts and other stakeholders for interviews and discussion were selected by the MOH Directorate of Communicable Diseases (DCD) and the MOA Chief Veterinary Officer. We selected three priority zoonotic diseases for our analysis with varying burdens on human and veterinary health sectors to better define nodes of communication and coordination as well as gaps for capacity building and systems strengthening. This type of analysis may identify current vertical, disease-specific strategies and frameworks that can be applied horizontally to develop national zoonotic disease strategies. It is important to note that our mapping does not address the role of livestock keepers and/or the density and number of livestock, which play a major role in disease outbreaks, transmission, and at times subsequent epidemics.

### Selection of priority zoonoses

There are multiple methods used in prioritizing disease detection and response capacity building, including analysis of the local and national burden of disease; global trends in emergence; economic costs associated and cross-sector impacts; human morbidity and mortality; and population health (3, 13–15). Our goal was to examine coordination and communications from the index case to notification at the national and international levels. In order to determine the mechanisms that promote and/or prevent information sharing across surveillance and laboratory networks both within and among ministries, it was first important to determine the priority zoonoses from both the public and veterinary health sectors. Both MOH and MOA have established priority notifiable disease lists, which are used to strengthen surveillance and laboratory capacities; however, there had not yet been a collaborative discussion on cross-linking these lists to develop formalized multi-sectoral priorities, particularly with respect to zoonotic diseases. We began with reviewing existing MOH and MOA notifiable disease lists and selecting the zoonotic diseases on each list for consideration. Through collaborative strategic discussions, we identified five MOH–MOA priority zoonoses for further ranking. We selected priority zoonotic diseases for case study analysis that aligned with three major categories of focus for intervention at the animal–human interface: endemic zoonoses, epidemic-prone zoonoses, and emerging zoonoses. Endemic zoonoses account for the majority of human cases and deaths, and the greatest reduction in livestock production. Epidemic-prone zoonoses occur sporadically or cyclically and the spatial distribution of outbreaks may vary, but epidemic-prone diseases are often prioritized due to their impact on health and trade. Emerging zoonoses (diseases that are either new to a population or are rapidly increasing in incidence or geographic range) generally account for only a fraction of the zoonotic disease burden, but outbreaks may have unpredicted and highly disruptive effects (16). We assigned weight to pathogens associated with a high human disease burden (morbidity and mortality); impact on livestock and wildlife (production, economic loss); amenability to practice- or veterinary medicine-based interventions; existing surveillance systems; and, finally, mechanisms for improved stakeholder communication and coordination (17–20).

### Mapping of surveillance and laboratory networks

**Figure 1:**
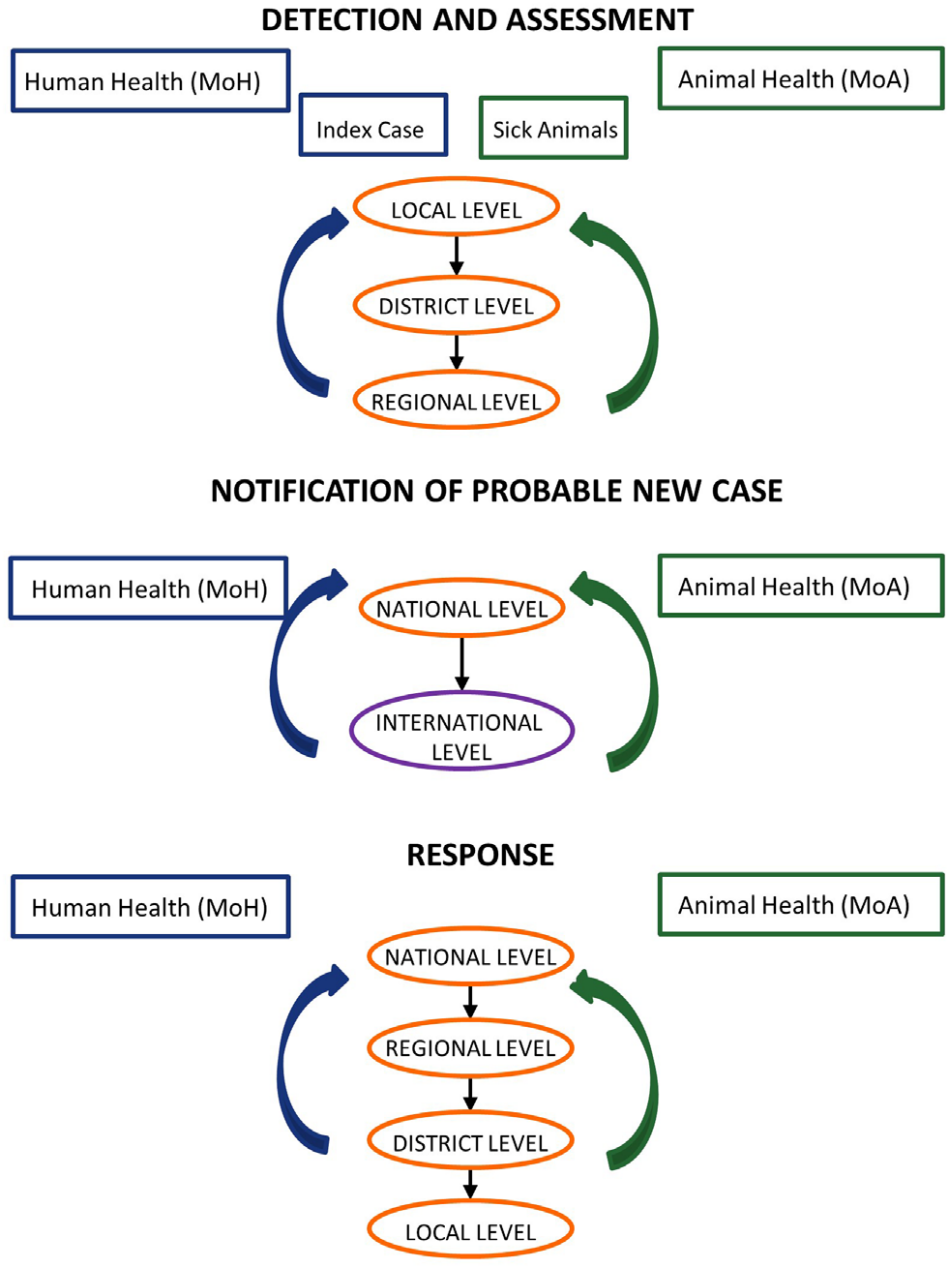
**Model joint assessment and response**. In collaboration with the Jordan Ministry of Health, Field Epidemiology Training Program, and Ministry of Agriculture, we examined coordination and communications from the index case to notification at the national and international levels in order to identify priorities and gaps that limit information sharing for actions. Efforts in surveillance and response lead by Ministry of Health are represented in blue while those lead by Ministry of Agriculture are in green.

**Figure 2:**
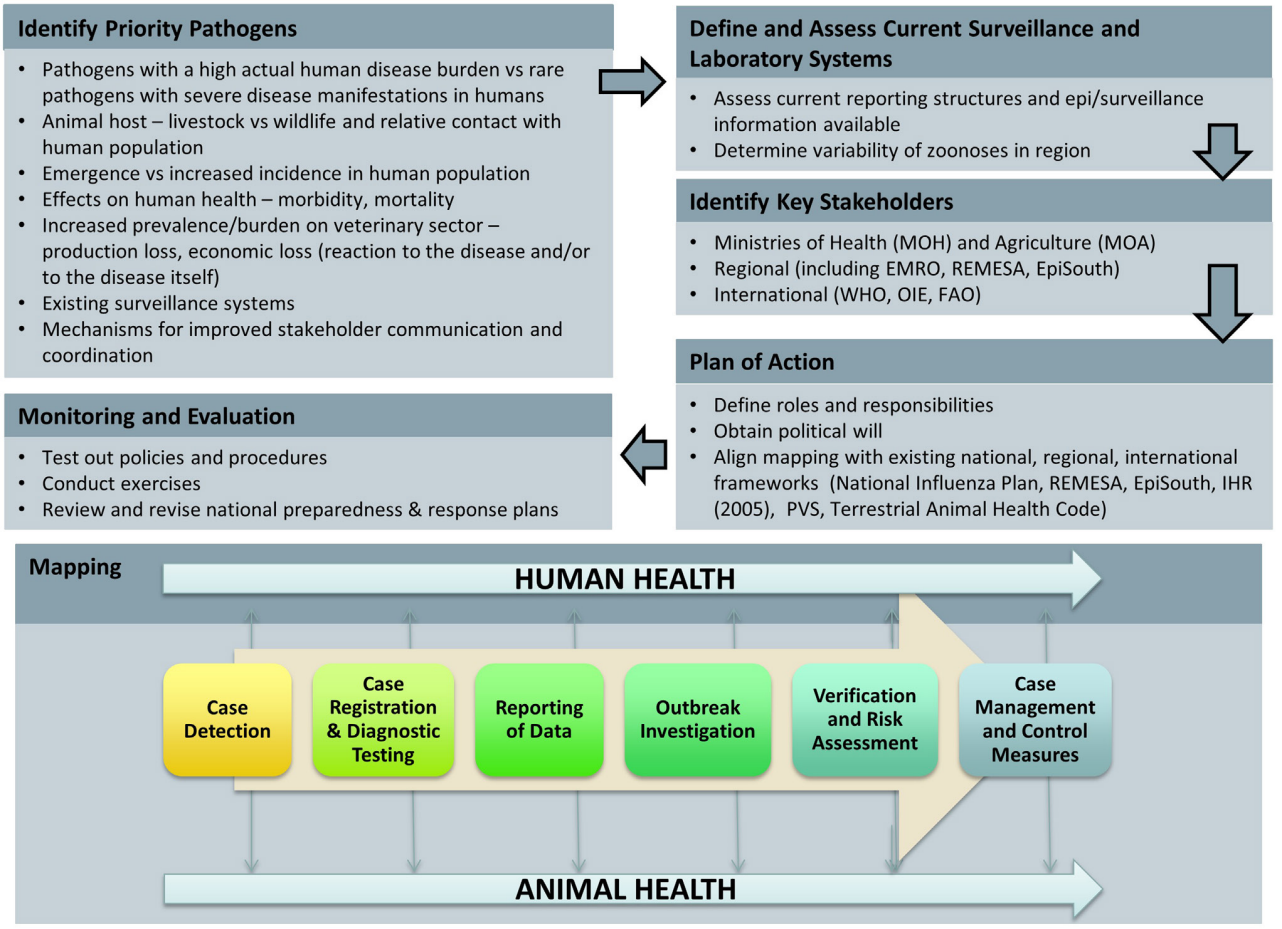
**Identifying priority zoonoses and identification and networks for case management**. In order to select three priority zoonotic diseases for analysis, we considered MOH and MOA notifiable disease lists as well as global priority zoonoses. For the three selected priority zoonoses, we developed case studies outlined in a five-step process: (1) case reporting; (2) reporting and sample submission; (3) laboratory testing; (4) case management; and (5) outbreak investigation. The resulting analysis is a systems map that identified the nodes of communication, coordination, and decision-making where the health and veterinary sectors intersect, highlighting both areas of strength and gaps that would benefit from capacity-building resources.

In collaboration with Jordan’s Field Epidemiology Training Program (FETP), we developed case studies based on past zoonotic events to examine coordination and communications from the index case to notification at the national and international levels, in order to identify priorities and gaps that limit information sharing for action (Figure 1). For the three selected priority zoonoses, we developed case studies outlined in a five-step process: (1) case reporting; (2) reporting and sample submission; (3) laboratory testing; (4) case management; and (5) outbreak investigation (Figure 2). For each case study, we created a decision tree at each of the steps noted above, identified the strengths and weaknesses of the system, and recommended steps for improvement. This resulted in a systems map that identified the nodes of communication, coordination, and decision-making where the human and veterinary health sectors intersect, highlighting areas of strength as well as gaps that would benefit from capacity-building resources. This information can be translated into recommendations for strengthening policies, protocols, and practices for preventing and responding to priority zoonoses across veterinary and public health sectors.

## Results

### Selecting priority zoonoses for analysis

In collaboration with the MOH-DCD and the MOA Veterinary Services, the combined Jordan FETP and The George Washington University Global Health Security Program (GWU) research team determined that the most suitable priority diseases for our analysis included HPAI H5N1, brucellosis, and rabies. These priority diseases represent endemic zoonoses (brucellosis), epidemic-prone zoonoses (rabies, defined as a disease in which exposures to a single infected animal can lead to multiple human cases) (16), and emerging zoonoses (HPAI H5N1).

### Mapping surveillance networks

The Ministry of Health is the largest financer and provider of health services in Jordan. Disease surveillance efforts in Jordan fall under the oversight of the Director of Primary Health Care Administration, which oversees eight directorates within MOH (21). The DCD within the Primary Health Care Administration is charged with disease surveillance and is most active in detection, surveillance, assessment, response, and reporting activities. Within DCD, the Surveillance Department, Division of Applied Epidemiology, and Division of Infection Control (among others) oversee specific programs and functions. DCD’s Surveillance Department receives and manages information from 22 surveillance sites throughout Jordan that track the 42 reportable diseases in country. Information flows from the health facility level to the health directorates, and then to DCD, where data are compiled and analyzed to prepare the weekly reporting bulletin. Within MOA, the Secretary General Assistant for Livestock and the Chief Veterinary Officer have responsibility for the organization and implementation of veterinary services, whereas the majority of administrative control falls to 13 agricultural departments. Veterinarians are trained in the field on zoonoses communication and reporting, sample collection, and packaging. Within the Veterinary Services Department, there is an Animal Health Section, Poultry Health Section, and Veterinary Quarantine Section, which coordinate with the governorate level departments on disease surveillance and response. Both MOH and MOA have notifiable disease lists for immediate, weekly, and monthly reporting.

### Mapping laboratory networks

Diagnostic and confirmatory laboratory services are provided from the Central Public Health Laboratory (CPHL) to the health center level. CPHL oversees laboratory biosafety and biosecurity programs for MOH laboratories and hospitals. Each health directorate has a laboratory coordinator at the governorate level. Although Laboratory Quality Management Systems (LQMS) and the logistical support to manage supplies and safe specimen transport exist they are uneven at the subnational level. Diagnostic and confirmatory testing capabilities are shared across public and private sector laboratories, which can provide challenges in the event of major outbreaks. CPHL coordinates with the U.S. Naval Medical Research Unit 3 (NAMRU-3) located in Cairo for confirmatory testing when necessary. MOA has veterinary laboratories in each of the 12 governorates that perform routine diagnostics at varying levels of capacity. A lack of resources, both human and financial, leads to a majority of diagnostic and confirmatory testing falling to the Central Veterinary Laboratory (CVL) (22). MOA coordinates with the UN Food and Animal Organization (FAO) and OIE to assist with confirmatory testing, as well as gold standard diagnostic tests when these are not locally available.

### Case study #1: Highly pathogenic avian influenza H5N1

As of 2006, Jordan and most of its neighbors have remained free of human HPAI H5N1 cases, with the exception of Egypt (which reported 48 deaths and 165 cases between November 2014 and April 2015) (23–25). Jordan’s geography puts it at low risk for the introduction of HPAI from migratory waterfowl due to its lack of surface water; key migratory bird habitats in the Jordan Valley and around the Gulf of Aqaba are distant from major poultry production facilities. A majority of Jordan’s poultry farms are commercial with backyard flocks comprising only 2% of the sector (22). The commercial sector is advanced for the region, including biosecurity into its best practices (26).

#### Existing Networks

Following devastating outbreaks of HPAI H5N1 in 2006, Jordan established the National Committees on Avian and Pandemic Influenza, including the National Steering Committee, the National Technical Committee, and the National Center for Security and Crisis Management (previously the Disaster Management Committee) each playing a role in detection, reporting, and response to highly pathogenic and pandemic influenza. Jordan has both an Animal Health National Preparedness Plan and National Contingency Plan for Avian Influenza, which are utilized by various ministries, including Ministries of Health, Agriculture, Planning, Foreign Affairs, Transport and Communication, Interior, Industry and Trade, Education, Communications and IT among others. At the regional level, the Middle East Consortium on Infectious Disease Surveillance (MECIDS) network developed an Avian and Pandemic Influenza Sub-Regional Common Plan of Action for Palestine, Jordan, and Israel^1^. The plan defines the protection zone (3 km radius from affected farm designated for culling), surveillance zone (10 km radius from affected farm where enhanced surveillance and control measures must be taken), and case definitions for avian and human influenza cases (suspected, probable, and confirmed). It also outlines principles, procedures, and protocols for MOA and MOH officials in the case of H5N1 in poultry (notification of suspected case, protection and surveillance zone established, lab confirmation of H5, follow-up) and in the event of H5N1 in humans (notification of suspected case, epidemiological investigation, lab diagnosis of H5 and follow-up). In 2008, 32 representatives from multiple sectors (health, transportation, education, interior, laboratory, and media) in Jordan, Palestine, and Israel participated in a regional pandemic influenza tabletop exercise to develop action items based on various influenza case scenarios, including human-to-human transmission of HPAI H5N1. This body is active in disease surveillance and response across a number of priority diseases for the region and is able to activate and respond in the event of HPAI if necessary. The 2006 HPAI H5N1 outbreak in poultry is a good example of how and when MOH and MOA communicate, particularly when there was an immediate need and financial resources.

#### Detection, Notification, and Response

**Figure 3:**
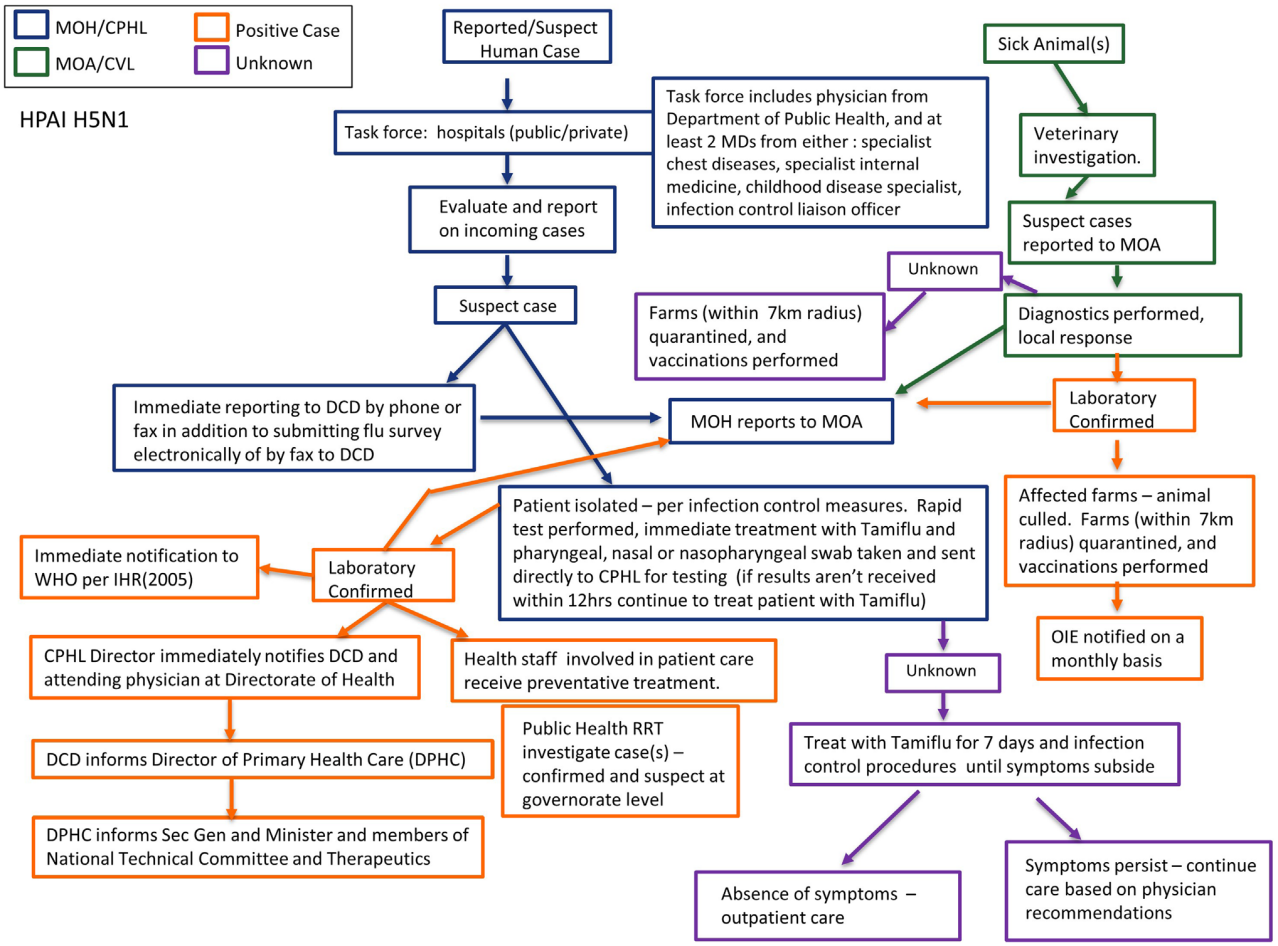
**Surveillance and laboratory mapping of highly pathogenic avian influenza (HPAI) H5N1**. Surveillance and laboratory networks for HPAI H5N1, including nodes of communication and response, were mapped across human health and veterinary sectors. Systems were analyzed beginning from report of a suspect case to diagnostic confirmation, including evaluation: case reporting; reporting and sample submission; laboratory testing; case management; and outbreak investigation. Efforts in surveillance and response lead by Ministry of Health are represented in blue while those lead by Ministry of Agriculture are in green. Positive cases are noted in orange while unknown/unconfirmed cases are represented in purple. Abbreviations: MOH, Ministry of Health; CPHL, Central Public Health Laboratory; DCD, Directorate of Communicable Diseases; MOA, Ministry of Agriculture; CVL, Central Veterinary Laboratory.

If a patient presents at a health facility or hospital and the clinician suspects HPAI based on clinical symptoms or due to reports of contact with sick poultry, the patient is isolated and samples are sent to the CPHL for diagnostic confirmation. The isolated patient is treated with antivirals and health care staff involved in patient care receives preventative treatment. HPAI is an immediate reportable Group A disease; the primary health care unit or hospital reports directly to the Health Directorate, which then reports to the DCD. MOH also communicates with MOA that there is a suspect human case of HPAI. Likewise, if there are reports of poultry deaths and/or an animal presents and is characterized as suspect HPAI, veterinary services will notify MOA, MOH, and collect samples for confirmation testing at the CVL. A positive rapid diagnostic test for type A influenza may result in quarantine or culling of affected farms while confirmation testing is performed at CVL. MOA reports positive cases to OIE on a monthly basis, whereas MOH would immediately report a positive human case as outlined under IHR (2005). If the CVL confirms HPAI, Rapid Response Teams (RRTs) assist in providing personal protective equipment (PPE) and restricting contact to affected farms/flocks to determine proper culling procedures. In addition, a poultry vaccination team will be deployed to farms/flocks within a 7-km radius. In the event of a confirmed human case public health RRTs will conduct in-depth reports and follow-up with possible suspect cases and contacts. If the patient’s symptoms persist with unconfirmed diagnosis, treatment with Tamiflu continues for 7 days and care is provided per physician recommendations. During an outbreak MOH and MOA will communicate laboratory confirmed cases to each other on a daily basis. Jordan has both an Animal Health National Preparedness Plan and National Contingency Plan for Avian Influenza, which are utilized by various ministries including MOA and MOH. Figure 3 depicts a flow chart schematic of surveillance and laboratory channels. Mechanisms for communication and coordination among laboratory, public health, and veterinary officials at the governorate and national level are strong in the event of a suspect case of HPAI H5N1. Frameworks and plans exist and function well; however, they are only activated in the case of emergencies.

### Case study #2: Rabies

Rabies is a zoonotic viral disease that causes acute inflammation of the brain in animals. The disease is spread to humans from another animal (e.g., dogs, camels, donkeys), commonly by a bite or scratch, although exposure of mucous membranes to infected saliva is also a risk. Globally, most cases are the result of a dog bite: exposure to rabid dogs is the cause of over 90% of human exposures to rabies and of over 99% of human deaths worldwide. Rabies is a completely preventable disease in the human population with effective veterinary vaccine campaigns and effective reporting and rapid post-exposure treatment following animal bites. More than 50,000 people die annually from rabies worldwide, despite the fact that the tools to prevent and manage the disease are readily available (27). Once clinical signs of rabies appear, the disease is nearly always fatal, and treatment is typically supportive.

#### Existing Networks

Human rabies cases are rather rare in Jordan. Dog bites account for the vast majority of suspect human rabies cases in Jordan (28). According to MOH, 4753 patients were treated for rabies exposure in 2013, but no human rabies cases were reported (or have been for the last 3 years). MOA reported a total of seven cases and seven deaths to OIE in 2013 (29). MOA provides free vaccines to vaccinate animals for prevention and control of rabies; however, there is a limited vaccine supply and an inability to cover the entire susceptible population. Currently, vaccine campaigns focus on the companion animal population, covering stray dogs only as supplies allow. There is no policy to vaccinate any potential wildlife reservoir. Key to the control of rabies in Jordan is the containment and vaccination of the stray dog population nationwide.

#### Detection, Notification, and Response

**Figure 4:**
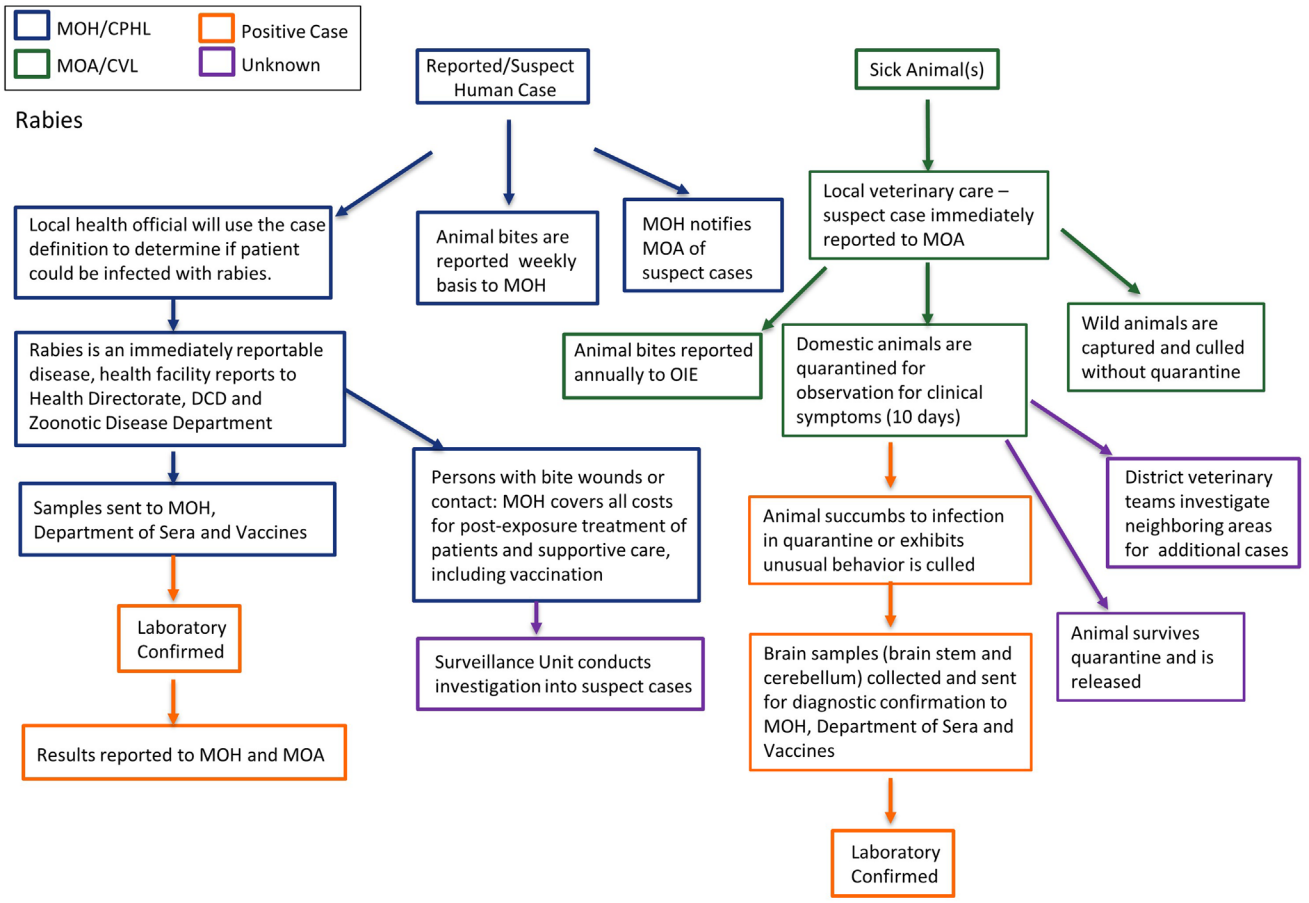
**Surveillance and laboratory mapping of rabies**. Surveillance and laboratory networks for rabies, including nodes of communication and response, were mapped across human health and veterinary sectors. Systems were analyzed beginning from report of a suspect case to diagnostic confirmation, including evaluation: case reporting; reporting and sample submission; laboratory testing; case management; and outbreak investigation. Efforts in surveillance and response lead by Ministry of Health are represented in blue while those lead by Ministry of Agriculture are in green. Positive cases are noted in orange while unknown/unconfirmed cases are represented in purple. Abbreviations: MOH, Ministry of Health; CPHL, Central Public Health Laboratory; DCD, Directorate of Communicable Diseases; MOA, Ministry of Agriculture; CVL, Central Veterinary Laboratory.

Any human bitten by stray or wild dogs is considered a probable rabies case and the responsible health official uses the case definition for determination. All suspect patients are treated post-exposure with the rabies vaccination and MOH covers all costs for post-exposure prophylaxis and supportive care. Patient samples are collected and sent to the Department of Sera and Vaccines for confirmation, however, testing of samples is not routine, which can lead to unnecessary costs of patient care from post-exposure prophylaxis for unconfirmed rabies cases. Rabies is an immediately notifiable disease, MOH notifies MOA of suspect cases; however, animal bites are reported to MOA on a weekly basis and to OIE annually. The Surveillance Unit within MOH conducts investigations into suspect cases and submit final reports to DCD. Occasionally, the RRT includes veterinarians and subject matter experts from MOA. In the event of suspect rabies case(s) in domesticated or wild animals, the local veterinary services is notified and if based on case definition the animal is labeled suspect, MOA is immediately notified for investigation. If the suspect case is in feral or otherwise non-domesticated animal(s), they are immediately culled without quarantine. If the animal(s) are domesticated, they are quarantined for 10 days under the observation of MOA; if the animal develops symptoms or succumbs to infection, samples are sent to MOH-Department of Sera and Vaccines for diagnostic confirmation. There is currently no public veterinary laboratory in Jordan that has capacity to diagnose rabies in animals. MOA will conduct an investigation of neighboring areas for additional cases and quarantine when necessary. Figure 4 shows a flow chart schematic of surveillance and laboratory channels. Key to the control of rabies in Jordan is the containment and vaccination of the stray dog population nationwide and timely confirmation of suspect human cases in order to prevent unnecessary extensive health care costs for post-exposure treatments on negative patients.

### Case study #3: Brucellosis

Brucellosis is an important zoonotic disease of livestock, notifiable to OIE (30). Globally, human brucellosis is a re-emerging zoonotic disease with an estimated 2% case fatality rate, even though successful eradication and control programs for domestic animals effectively and significantly decrease disease incidence in humans, and have been established in many at-risk countries. Symptoms of brucellosis in humans include fever with multiple non-specific clinical signs and symptoms. Delayed diagnosis, chronic disease, failure of primary antibiotic treatment, and relapses are common. Brucellosis is transmitted through exposure to infected animal products (most commonly raw dairy products) or, less frequently, through direct contact with infected camels, cattle, sheep, or goats. More than 500,000 human cases are reported worldwide each year, (31) but the number of undetected cases is believed to be considerably higher. *Brucella* spp. are also categorized as potential biological agents for deliberate use in many US and international frameworks due to their high contagiousness and their impact on human and animal health.

#### Existing Networks

In 1985, an official system for reporting human cases of brucellosis was established by MOH, under the supervision of the Communicable Diseases Control Program Division. Spurred by a large brucellosis outbreak in Jordan roughly 10 years ago, the MOH’s Division of Zoonotic Diseases and veterinary public health actors at the MOA developed a cooperative relationship in reporting and response to brucellosis. However, there is no national plan. According to OIE reporting, brucellosis continues to be in the top three zoonotic diseases reported in Jordan (29). In collaboration with CDC, the MOH and others conducted a burden of illness study in 2003, including population, animal vaccinations, and laboratory surveys and validation study. However, outbreaks are still prevalent in Ma’an and Mafraq governorates on a seasonal basis and for various reasons, including the lack of clear clinical symptoms and misdiagnosis, human brucellosis is significantly under-reported and under-diagnosed, particularly by the private health sector (32, 33). In Jordan, ruminants, particularly sheep and goats, are vaccinated at all ages, at any time during the year, and annual revaccination is recommended. On average, about 18–25% of the sheep and goats in Jordan were vaccinated through 2000, although unofficial estimations on vaccine coverage is increasing and can be estimated at times to be as high as 50% recently published data indicates that only 1.5% of the small ruminant population is vaccinated leading to regional endemicity, particularly in the north (34, 35). Starting in 2015 a new project will begin, a partnership between EMPHNET and CDC with Jordan MOH as lead implementer, to estimate disease burden in the human population (36).

#### Detection, Notification, and Response

**Figure 5:**
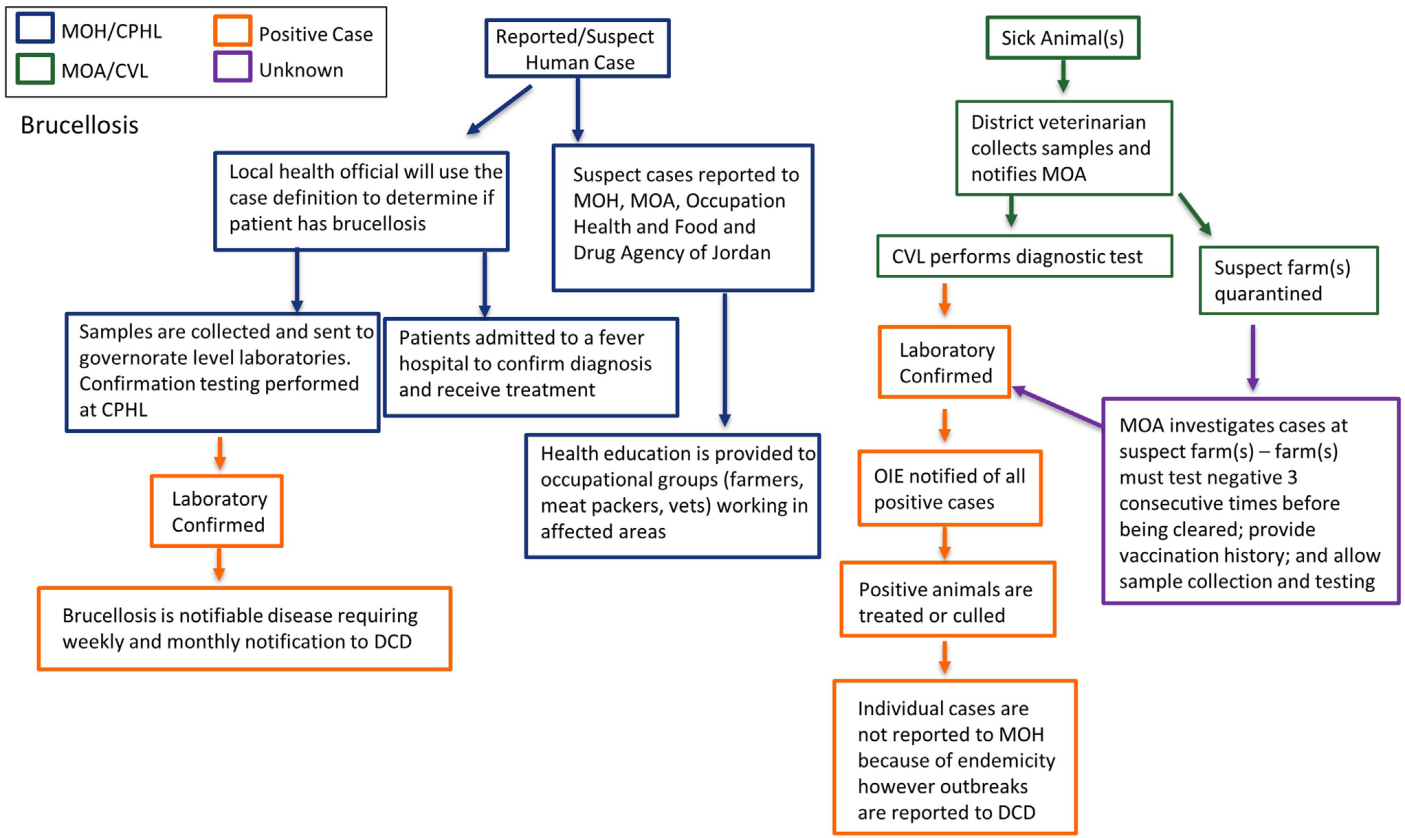
**Surveillance and laboratory mapping of brucellosis**. Surveillance and laboratory networks for brucellosis, including nodes of communication and response, were mapped across human health and veterinary sectors. Systems were analyzed beginning from report of a suspect case to diagnostic confirmation, including evaluation: case reporting; reporting and sample submission; laboratory testing; case management; and outbreak investigation. Efforts in surveillance and response lead by Ministry of Health are represented in blue while those lead by Ministry of Agriculture are in green. Positive cases are noted in orange while unknown/unconfirmed cases are represented in purple. Abbreviations: MOH, Ministry of Health; CPHL, Central Public Health Laboratory; DCD, Directorate of Communicable Diseases; MOA, Ministry of Agriculture; CVL, Central Veterinary Laboratory.

When a patient presents with symptoms consistent with brucellosis and has ingested raw milk or other potentially infected dairy products, the health official will use the case definition to determine whether to classify the case as suspect brucellosis. Suspect human cases are reported to MOH and the Occupational Health and the Food and Drug Agency of Jordan. Patients may be admitted to a fever hospital to confirm diagnosis and initiate treatment. Clinical samples are sent to the governorate level laboratory for initial diagnostic testing, and to CPHL for confirmatory testing as indicated. The lab results are not shared with MOA. When possible, health education is provided to at-risk occupational groups (farmers, meat packers, dairies) working with animals or animal products; however, there is no clear guidance for surveillance and outbreak response for MOH. In the event of a suspect case or farm(s), the local veterinary services will quarantine the suspect farm(s) and collect samples for diagnostic testing at the CVL, at times and when possible governorate level labs will perform diagnostics. A team is sent to each suspect farm to conduct an investigation, which includes an imposed quarantine, provision of herd vaccination history, sample collection, and testing. A farm must test negative three consecutive times before being cleared. Any animals testing positive must be culled. It should be noted that this is the recommended procedure; however, we do not have country-wide data as to whether this is implemented. MOA reports all positive cases to OIE. Individual animal cases of brucellosis are not reported to MOH due to the endemicity of brucellosis in Jordan; however, outbreaks are reported directly to DCD. Please see Figure 5 for a flow chart schematic of surveillance and laboratory channels. As noted above, effective livestock vaccine campaigns can significantly reduce the burden of human brucellosis. There are clear seasonal patterns associated with human cases and outreach and education on zoonotic transmission will be key in containing human outbreaks.

## Discussion

Mapping of zoonoses and the burden of such diseases can help identify vulnerabilities not only where zoonoses pose significant health threats but also where efforts can be focused to improve prevention, communication, and coordination across veterinary and human health. These study findings describe existing systems that can be strengthened or applied by stakeholders to address current needs within Jordan, and offer case studies that can be applied in other contexts. Although the findings may appear predictable to those already deeply familiar with Jordan’s surveillance and response systems, the formal linkages within and across sectors may not be immediately obvious to the increasingly diverse stakeholder and partner networks engaged in long-term capacity building for global health security.

**Figure 6:**
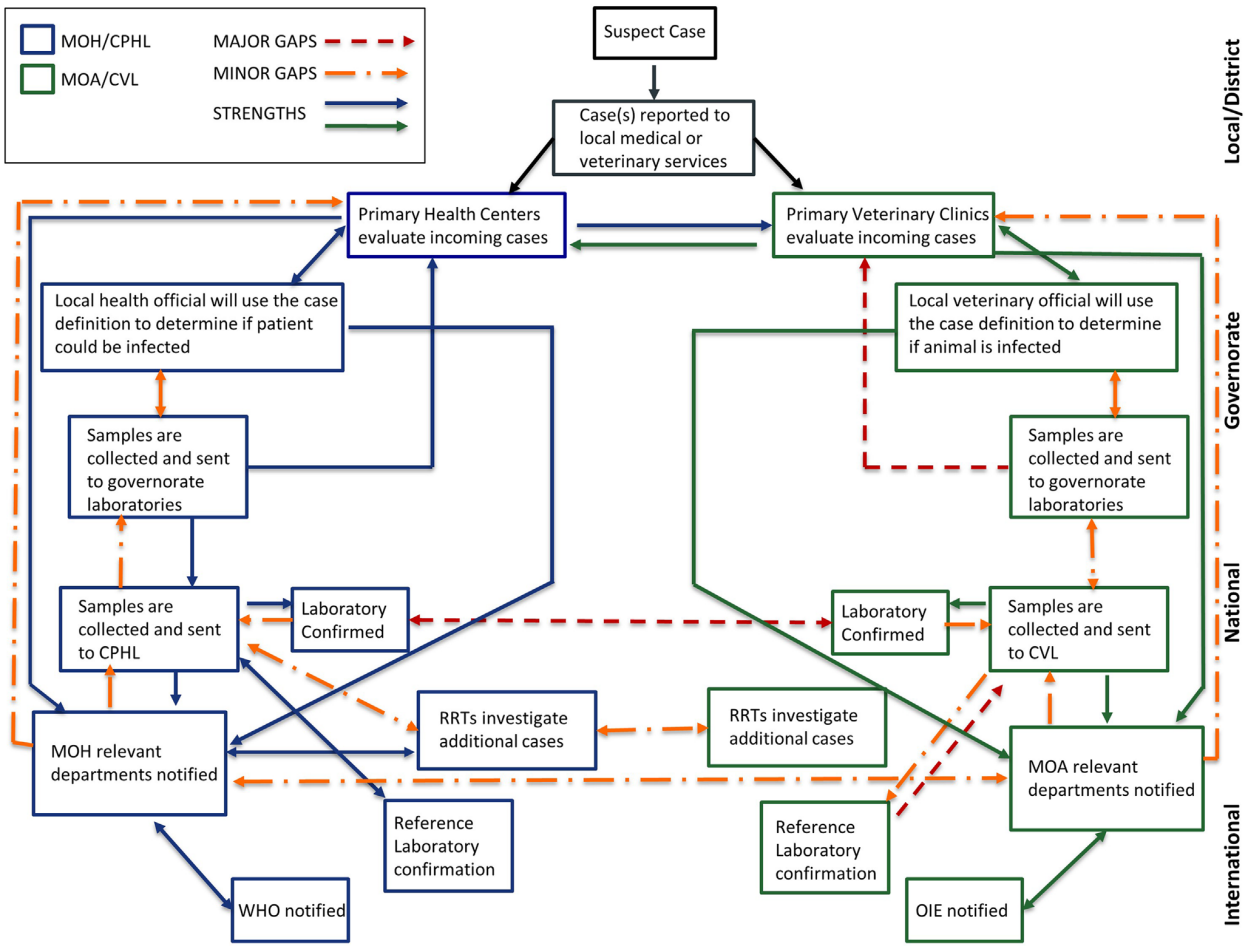
**Mapping public health and veterinary surveillance and laboratory networks**. An overall analysis of existing surveillance and laboratory networks for zoonotic diseases, including nodes of communication and response, were mapped across human health and veterinary sectors to indicate areas of strength and those requiring strengthening. Efforts in surveillance and response lead by Ministry of Health are represented in blue while those lead by Ministry of Agriculture are in green. Strengths within and across sectors are represented by solid blue and green lines. Major gaps are represented in red while minor gaps are represented in orange. Abbreviations: MOH, Ministry of Health; CPHL, Central Public Health Laboratory; DCD, Directorate of Communicable Diseases; MOA, Ministry of Agriculture; WHO, World Health Organization; OIE, World Organisation for Animal Health; RRTs, Rapid Response Teams.

We found many similarities in surveillance and response capacities across local, governorate, and national public and veterinary health networks regardless of the pathogen mapped, indicating that improvement in response to one specific pathogen would most likely improve the ability to respond to other zoonoses (Figure 6). The results of our mapping highlighted three main areas for improvement toward building national One Health capacities: (1) a national zoonotic reporting and communication framework, (2) a national zoonotic preparedness and response plan, and (3) increased laboratory diagnostic capacity across governorate level laboratories.

### National zoonotic reporting and communication framework

There are strong informal mechanisms for communication and coordination within and across local public health and veterinary services with consistent reporting up to governorate and national levels. However, the local facilities are not always involved in outreach and communication strategies for local response. There is no standardized structure for communication and information sharing across and within surveillance sectors and laboratories. There is no formal mechanism or protocol for reporting laboratory confirmation beyond CPHL and CVL obligations to report back to their relevant ministry departments. There is little, if any, cross-talk between CPHL and CVL in both surveillance reports and laboratory confirmation. This node of cross-sector communication is of particular importance when considering sentinel and early warning systems for zoonotic disease outbreaks in the veterinary sector and in investigations and response during simultaneous outbreaks of zoonoses in humans and animals. We recommend establishing a framework for reporting and communication to and from ministry department focal points to their local and governorate counterparts as well as across sectors at each level of reporting.

### National zoonotic preparedness and response plans

Rapid response teams, both locally and nationally deployed, are effective in outbreak investigation within their respective sectors; however, organization and deployment of multi-disciplinary RRTs are extremely pathogen dependent. This inconsistency can lead to duplication of efforts during critical phases of outbreak response and containment. Although there are disease-specific plans, such as the Animal Health National Preparedness Plan and National Contingency Plan for Avian Influenza, no national framework for preparedness and response to priority zoonotic diseases exists. We recommend that RRTs should be multi-disciplinary at the national level, using the FETP as resource to link governorate level epidemiologists available for rapid response.

### Laboratory capacity

Local and governorate level public health and veterinary laboratory capacity is inconsistent. Some labs lack the ability to perform routine diagnostics, due either to constraints in infrastructure, equipment, human resources, and/or funding. This inconsistency leads to delays in time to pathogen confirmation and response as well as increased diagnostic burdens on the national level laboratories, and at times, outsourcing to private laboratories for diagnostic confirmation. We propose that Jordan develop a national laboratory network, modeled after their experience as a member of the Network for the Control of Public Health Threats in the Mediterranean Regional and South East Europe (EpiSouth) Laboratory Network, to provide a formalized, standard protocol for private and public laboratory partnership for diagnostic testing or priority pathogens in the event of public and veterinary health events and those for routine testing for sentinel surveillance efforts.

Although this project focused on three priority zoonotic diseases in Jordan the challenges identified from both public health and veterinary surveillance and laboratory sectors are challenges faced by many middle income countries. Our analysis indicates that the HPAI networks in Jordan are well developed, coordinated, and effective in event identification, diagnosis, and response, which suggests that these existing resources can and should be leveraged to develop a comprehensive laboratory and surveillance One Health network.

## Author Contributions

ES, SK, JF, and RK conceived of the research; MA, NM, SK, CS, ES, and IB conducted the research; MA, NM, SK, ES, IB, and JF wrote the manuscript; CS and RK provided technical review and input to the manuscript.

## Conflict of Interest Statement

The authors declare that the research was conducted in the absence of any commercial or financial relationships that could be construed as a potential conflict of interest.

## Footnotes

^1^http://www.mecidsnetwork.org/
